# Polyphenol- and Glucuronoxylan-Rich Fiber Extract from Birch (*Betula* sp.) Wood Regulates Colonic Barrier Function and Cell Proliferation in Healthy Rats

**DOI:** 10.1021/acs.jafc.3c07757

**Published:** 2024-02-12

**Authors:** Emma Kynkäänniemi, Jere Lindén, Suchaya Ngambundit, Laura A. Saarimäki, Dario Greco, Hana Slaba, Maarit H. Lahtinen, Kirsi S. Mikkonen, Anne-Maria Pajari

**Affiliations:** † Department of Food and Nutrition, University of Helsinki, 00014 Helsinki, Finland; ‡ Department of Veterinary Biosciences, and Finnish Centre for Laboratory Animal Pathology (FCLAP), Helsinki Institute of Life Science (HiLIFE), University of Helsinki, 00014 Helsinki, Finland; § Finnish Hub for Development and Validation of Integrated Approaches (FHAIVE), Faculty of Medicine and Health Technology, Tampere University, 33520 Tampere, Finland; ∥ Division of Pharmaceutical Biosciences, Faculty of Pharmacy, University of Helsinki, 00014 Helsinki, Finland; ⊥ Helsinki Institute of Sustainability Science (HELSUS), University of Helsinki, P.O. Box 65, Helsinki 00014, Finland

**Keywords:** intestinal barrier, mucus layer, birch (Betula sp.), tight junctions, lignin, artificial intelligence (AI)

## Abstract

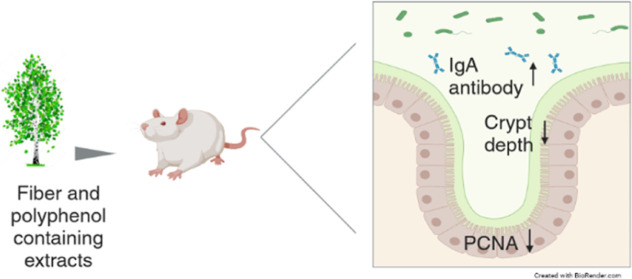

Birch wood-derived fiber extracts containing glucuronoxylans (GX) and polyphenols show potential for various food technological applications. This study investigated the effect of two extracts, GXpoly and pureGX, differing in lignin content on colonic barrier function. Healthy rats were fed diets containing 10% GXpoly, pureGX, or cellulose for 4 weeks. Colon crypt depth was lower in the GX groups than in the control group, but in the proximal colon, the result was significant only in GXpoly. An artificial intelligence approach was established to measure the mucus content and goblet cells. In the distal colon, their amounts were higher in the control group than in the GX groups. All diets had a similar effect on the expression of the tight junction proteins occludin, claudin-1, and claudin-7. GXpoly enhanced the fecal IgA production. Our results suggest that GX-rich extracts could support the colonic barrier and work as functional food ingredients in the future.

## 1 Introduction

Accumulating evidence suggests that dietary modulation can elicit both beneficial or harmful effects on the intestinal barrier.^1^ The barrier encompassing structural and functional characteristics, such as the mucus layer, epithelial cells, paracellular barrier, and immune responses, plays a crucial role in maintaining gut health.^1^ Polyphenols and dietary fiber have an important role in maintaining a healthy intestinal barrier; however, as their usual dietary intake is low, new sources that can be easily incorporated into foods are needed.

In the colon, diets rich in dietary fiber and polyphenols have been shown to improve different elements of the intestinal barrier.^1^ For example arabinoxylan, an abundant dietary fiber and a component of the plant cell wall of various grains, was able to increase the number of mucus-producing cells and mucosal secretory immunoglobulin A (sIgA) concentration in piglets.^2^ Xylo-oligosaccharides, which can be derived from xylans, helped to maintain the crypt cell integrity and increased tight junction protein expression in murine colitis models.^3^ However, dietary fibers with different physicochemical properties have been shown to have divergent effects on the components of the intestinal barrier.^4^

Polyphenols, found in plants and organisms, such as algae and edible mushrooms, are the most common bioactive components in the human diet. Lignin is the most abundant polyphenolic compound in whole-grain- and wood-derived extracts.^5^ A diet containing lignin-rich wheat bran was able to support intestinal integrity in weaned piglets^6^ and enhance intestinal IgA production in mice.^7^ However, studies addressing the effects of lignin or the synergic impact of lignin and dietary fiber on the colonic barrier are scarce. In addition, as lignin is a heterogeneous polymer and its final structure depends on the processing method,^8^ the impact of differently sourced lignin on gut health may vary.

Intestinal barrier dysfunctions have been associated with several chronic conditions,^1^ which has led to intensified interest to develop new methodological approaches to study intestinal barrier function. While blood biomarkers are usually used to assess intestinal permeability in vivo,^9^ emerging technologies such as deep learning artificial intelligence (AI) can be used to analyze histological samples and get more tangible information on the intestinal barrier. In recent years, it has been applied to digital pathology, especially in diagnostic-, prognostic-, and treatment prediction-related tasks in cancer. Even though using AI in the analysis of histological samples improves the time-efficacy and objectiveness and makes it easier to measure the intestinal barrier from whole tissue samples, the method has not been widely used to measure healthy tissues.

We studied the pivotal role of polyphenols and dietary fiber in maintaining a healthy intestinal barrier using birch-wood-derived fiber known as glucuronoxylan (GX). GX is an abundantly available byproduct of the forest industry and has the potential to serve as an economical and sustainable multifunctional food stabilizer and antioxidant.^10^ Moreover, in our previous study, birch GX- and lignin-rich extracts supported the growth of potentially beneficial bacteria and bacterial metabolites such as short-chain fatty acids (SCFA).^11^ These properties make GX a promising candidate as a novel dietary fiber. However, the impact of GX and residual lignin in GX extracts on the colonic barrier is not known.

Given that the physiological effects of dietary fiber can be influenced by the source and processing, the impact of birch GX on the colonic barrier cannot be directly deduced from the studies with other xylans. In addition, unlike studies conducted in disease models, we focus on unchallenged healthy animals to provide insights into the potential mechanisms underlying the effects of birch-derived fibers and polyphenols on colonic health. By comparing the effects of two diets (GXpoly and pureGX) containing soluble GX to a diet containing cellulose, a commonly used fiber in semisynthetic animal diets, on mucus volume, tight junction protein expression, immune function markers, and colon cell proliferation in healthy Wistar rats, the aim was to elaborate the mechanisms of GX-rich extracts on the intestinal barrier function and its potential as an intestinal health-promoting dietary fiber.

## 2 Materials and Methods

### 2.1 Glucuronoxylan-Rich Fiber Extract

The GX- and polyphenol-rich extract (GXpoly) from the Natural Resources Institute Finland (Luke) was prepared via pressurized hot water extraction, according to Kilpeläinen et al.,^12^ and spray dried. The highly purified GX-rich extract (pureGX) from CH-Bioforce Oy was recovered as a xylan concentrate (20% w/w) using the BLN process^13^ and precipitated with ethanol. The monosaccharide composition and acetyl groups of hemicelluloses have been previously analyzed by Mikkonen et al.^14^ The monosaccharide composition of GXpoly was β-d-xylopyranosyl 81%, 4-*O*-methyl-α-d-glucopyranosyl uronic acid 6.3%, β-d(1 → 4)-mannopyranosyl 3.9%, and α-d(1 → 6)-galactopyranosyl 2.2%, while pureGX had β-d-xylopyranosyl 79%, 4-*O*-methyl-α-d-glucopyranosyl uronic acid 7.3%, α-d(1 → 6)-galactopyranosyl 4.1%, and β-d(1 → 4)-mannopyranosyl 2.9%. Other carbohydrates were present in both extracts in minor quantities. The degree of acetylation was 55% in GXpoly and 51% in pureGX.

Lignin contents of extracts and diets were previously measured with pyrolysis-gas chromatography/mass spectrometry.^11^ Analysis showed that the GXpoly extract contained more lignin than pureGX [12.22 ± 2.16 syringyl and 5.81 ± 0.10 guaiacyl units (%) in the GXpoly extract compared to 1.72 ± 0.42 syringyl and 0.62 ± 0.21 guaiacyl units (%) in the pureGX extract]. However, lignin was only detected (concentration was below the detection limit) in the samples from the GXpoly diet, not in the samples from the pureGX diet.

### 2.2 Animals and Diets

The animal study protocol was approved by the Finnish National Animal Experiment Board (Eläinkoelautakunta, ELLA; permit code: ESAVI/12806/2019), and the research was carried out according to ARRIVE guidelines.^15^ The details of the rat feeding design and diet preparation are reported elsewhere.^11^ Briefly, 4 weeks old female and male Wistar (RccHan: WIST) rats purchased from Envigo (Horst, The Netherlands) were acclimatized for 12 days (diet SDS RM1, Special Diets Services, Witham, UK) and then fed pelleted, modified AIN-93G diets (Envigo Teklad Diets, Madison WI, The United States) for 4 weeks. The modified diets contained 10% (w/w) cellulose (control group), 10% GXpoly, or 10% pureGX (Table S1).

Before the feeding period, the rats were randomized into three body weight-matched groups, 14 rats (seven males and seven females) in every dietary group and housed in groups of 3 or 4 rats per cage. After the 4 weeks feeding period, they were killed by CO_2_ inhalation followed by cervical dislocation. Sacrifice order was evenly distributed among dietary groups over 7 days. After cervical dislocation, blood was drawn from the abdominal aorta, and the colons were collected from the anus to the cecum on a cold plate. The colons were divided into distal (the part closer to the anus), mid-, and proximal (the part closer to the cecum) segments. Segments were further handled according to their intended use.

### 2.3 Colon Histology

For histological analysis, a 5 mm transversal segment from the proximal end of the proximal and distal colon samples was collected with a fecal pellet inside. A distal segment from the proximal and distal colon was opened along the longitudinal axis, and fecal pellets were collected, segment rinsed with saline, and rolled into a Swiss roll with a toothpick starting from the proximal end.

The samples were fixed for 15 h in 4% buffered paraformaldehyde (Swiss rolls) or in Carnoýs solution (transversal samples).^16^ After fixation, the paraformaldehyde-fixed Swiss rolls were transferred into 70% ethanol until routinely processed and embedded in paraffin. The Carnoy’s solution -fixed transversal samples were rinsed 3 × 60 min at 37 °C in anhydrous ethanol, transferred to a tissue processor, and kept 2 × 60 min in xylene and 3 × 60 min in paraffin at 60 °C until embedding. Section thickness was 4 μm for both histochemical stains and immunohistochemistry. Hematoxylin and eosin (H&E) stain was used for measuring crypt architecture and periodic acid-Schiff–Alcian blue (PAS–AB) stain to identify goblet cells and mucus.

### 2.4 Imaging, Image Acquisition, and Analysis

The colon sections were digitalized with bright-field scanning using Pannoramic 250 scanner (3DHISTECH, Budapest, Hungary) at 20× magnification, 0.24 μm/pixel resolution, and 20*×*/0.8 NA objective.

Two independent researchers measured the depth of the crypts from the Swiss rolls using SlideViewer 2.5 (3DHISTECH, Budapest, Hungary). All the criteria fulfilling crypts (avg. 14 per image) were measured from the H&E-stained proximal and distal colon samples. Acceptable crypts had a discernible basement membrane and visible single layer of epithelial cells to trace the crypt from the base to the top. In addition, crypts had to be straight enough so that a straight line could be drawn from the bottom of the crypt to its surface.

The amount of mucus volume and the number of goblet cells were analyzed with an Aiforia image management and analysis platform (Aiforia Technologies, Helsinki, Finland). The AI model for goblet cell analysis was developed using 54 representative images (65%, *n* = 83). For goblet cell counting, the AI model was taught to recognize crypts having a visible basement membrane and be traceable from laminal propria until the surface of the epithelium. Approximately 80% of the crypt had to be visible. Basal and apical parts of the crypts were analyzed separately to differentiate goblet cells from mucus-secreting epithelial cells and to investigate possible changes in the goblet cell distribution. AI was taught to separate PAS- and AB-stained cells and cells that were not clearly red (PAS) or blue (AB). For mucus volume analysis, only whole transversal samples were used (*n* = 70, 37 distal, and 33 proximal). AI was trained to measure the colonic mucus layer area using 23 representative images (33%). Mucus that was inside the goblet cells or penetrated the fecal pellet was not included in the analysis.

### 2.5 Immunohistochemistry

Claudin-7 and ZO-1 were localized in Carnoy’s solution fixed transversal proximal (*n* = 5) and distal (*n* = 5) colon samples. For ZO-1, antigen retrieval was conducted in 0.01 M Tris–EDTA, pH 9.0 at 99 °C for 20 min, while for Claudin-7, no pretreatment was used. After antigen retrieval, the sections were washed in TBS buffer with 0.5% Tween, endogenous peroxidase blocked with 3% hydrogen peroxide in PBS, and nonspecific antibody binding with 10% BSA in PBS. Primary polyclonal rabbit antibodies, anticlaudin-7 (1:400; Invitrogen, Thermo Fisher Scientific, Waltham, MA, United States) and anti-ZO-1 (1:600; Invitrogen), were incubated for 60 min at room temperature. The secondary HRP-linked antibody (BrightVision + Poly-HRP kit; ImmunoLogic, Duiven, The Netherlands) was incubated at room temperature for 30 min, and the immunoreaction was visualized with the Bright DAB Substrate kit (ImmunoLogic). The tissue sections were photographed using an Axio Lab A1 microscope equipped with an Axiocam 305 color camera (Carl Zeiss, Jena, Germany).

### 2.6 Western Blot

For protein extraction, scraped mucosal tissue segments (15 mm) from the distal end of proximal and distal colon frozen in liquid N_2_ were homogenized with TissueLyser II (Qiagen, Hilden, Germany) in 300 μL lysis buffer [1 M Tris–HCl (Sigma-Aldrich, St. Louis, MO, USA), 1 M NaCl, 0.1 M EDTA (Millipore, Burlington, MA, USA), 20% (w/v) Triton X-100 (Sigma-Aldrich), 0.1% (w/v) SDS, 0.5 M NaF, 1 M Na_3_VO_4_, and protease inhibitor cocktail (Thermo Fisher Scientific)]. The homogenate was centrifuged (SL 8R, Thermo Fisher Scientific) at 10 000 rpm at 4 °C for 5–7 min. The protein concentration of the supernatant was measured with a Bradford assay (SkanIt Software 6.0.2.3, Thermo Fisher Scientific). The samples were stored at −20 °C until further use.

The samples were mixed with protein sample loading buffer (LI-COR Biosciences) and heated at 98 °C for 5 min. Subsequently, 40 μg of protein from each sample and 5 μg of positive control (A431 Cell Lysate, BD Transduction Laboratories, Franklin Lakes, NJ, USA) were loaded onto a 4–12% Bis–Tris SDS-PAGE minigel (Invitrogen) and run for 45 min at a constant voltage of 180 V. The proteins were transferred onto a polyvinylidene fluoride membrane for 1 h at 100 V. For normalization, the blots were stained (Revert 700 Total Protein Stain and Wash Solution Kit, LI-COR Biosciences, Lincoln, NE, USA) according to the manufacturer’s instructions and then blocked with a blocking buffer [Intercept (TBS) Blocking Buffer, LI-COR Biosciences] for 1 h at room temperature to prevent nonspecific binding.

The blots were incubated with polyclonal rabbit-antioccludin (1.5:1000; Invitrogen) and monoclonal mouse-anticlaudin-1 (1:1000; Invitrogen) overnight at 4 °C. The blots were then washed with TBS and incubated with goat antirabbit (1:10 000; LI-COR Biosciences) and goat antimouse secondary antibodies (1:10 000; LI-COR Biosciences) for 1 h at room temperature. Immunofluorescent bands of occludin and claudin-1 were detected and quantified before stripping and reprobing for claudin-7. The stripped blots were incubated with polyclonal rabbit-anticlaudin-7 (1:1000; Invitrogen) overnight at 4 °C and subsequently washed and incubated with goat antirabbit secondary antibody (1:10 000; LI-COR Biosciences) for 1 h at room temperature. PCNA was analyzed using PCNA polyclonal antibody (1:1000; Proteintech Group, Inc., Rosemont, IL, USA) and goat antirabbit secondary antibody. All immunofluorescent bands were detected, and the band intensities were quantified with an Odyssey Classic Imaging System (LI-COR Biosciences).

### 2.7 ELISA

The fecal IgA concentration and serum lipopolysaccharide binding protein (LBP) concentration were analyzed with ELISA. Before the IgA analysis, 20 mg of lyophilized rat fecal sample from the proximal colon was mixed with 0.5 mL of PBS containing protease inhibitor cocktail (Thermo Fisher Scientific). Samples were homogenized (TissueLyser II, Qiagen) with two 2 mm steel beads and centrifuged (SL 8R, Thermo Fisher Scientific) at 16 000 g at 4 °C for 10 min, after which the collected supernatant was quantified by ELISA according to manufacturer’s instructions (Cusabio, Houston, TX, USA). Serum LBP was analyzed with ELISA according to manufacturer’s instructions (Cusabio). The optical density of each well was measured at 450 nm and corrected with subtracting readings at 540 nm using a microplate photometer (Multiskan FC, Thermo Fisher Scientific).

### 2.8 RNA Isolation and Gene Expression Analysis

For RNA extraction, the mucosa of colon mid-segment (2 cm) was collected using a microscope slide and stored in RNALater for 24 h at +4 °C, after which it was transferred to −20 °C. Total RNA was extracted with the TRIzol Plus RNA Purification Kit (Invitrogen) according to manufacturer’s instructions. The extracted RNA was quantified, and its purity was assessed using a Nanodrop 1000 spectrophotometer (Thermo Fisher Scientific), and RNA’s integrity was confirmed with Bioanalyzer 2100 (v2.6; Agilent, Santa Clara, CA, USA). In total, 32 samples were used in microarray analysis with RIN (an RNA integrity number) > 8 and purity between 1.97 and 2.16 using the A260/A280 ratio.

Samples were analyzed using SurePrint G3 Rat Gene Expression v2 8 × 60K Microarray (Agilent) following the manufacturer’s instructions. Briefly, 200 ng of total RNA was used to synthesize cDNA, which was further transcribed into cRNA (Low Input Quick Amp Labeling Kit, no-dye, Agilent). cRNA was labeled with nucleotides containing either Cy3 or Cy5 fluorescent labels (Agilent) and then purified using the RNeasy Mini Kit (Qiagen). The quantity and specific activity of cRNA was checked with NanoDrop (ND-2000, Thermo Fisher Scientific). Two samples with different labels were randomly combined to form the microarray slides. Slides were hybridized for 17 h, after which they were washed and scanned with the Agilent microarray scanner model G2505C. Normalized and raw microarray data are available at Gene Expression Omnibus (GEO) at the National Center for Biotechnology Information (NCBI). The GEO accession number is GSE230139.

The microarray data were preprocessed and analyzed with R Shiny application eUTOPIA.^17^ In the quality check, one array was removed, after which 30 samples were included in the final analysis. The raw data were normalized using quantile normalization method and dye; array and slide associated variation was corrected using the method ComBat from R package sva.^18^ Differentially expressed genes were identified by linear models, and pairwise comparisons were performed with empirical Bayes using the R package limma.^19^ Pairwise comparisons were done between pureGX vs Control, GXpoly vs Control, and pureGX vs GXpoly using corrected batches and sex as covariate. A sparse partial least-squares-discriminant analysis (sPLS-DA) was performed to visualize the classification of the gene expression between the GXpoly, the pureGX, and the control group using the mixOmics R package.^20^

### 2.9 Statistics

Before the statistical analysis, data were checked for non-normal residual distribution, heterogeneity of variance, and outliers. The data were analyzed with ANOVA using sex as a factor. Variables that did not meet the assumptions of parametric tests (IgA, proximal colon occludin, and distal colon occludin, claudin-1, and claudin-7) were log transformed. The Tukey HSD was used for pairwise comparisons. For analyzing western blot results, the blot was used as a random factor. Results from histological samples were analyzed using a nonparametric test (Kruskall–Wallis test), and the Dunn-Bonferroni approach was used for pairwise comparisons. Results are reported as mean ± standard deviation, and *p*-value ≤ 0.05 was considered significant. Analyses were conducted with IBM SPSS Statistics 28.

## 3 Results

### 3.1 General Observations

Animal-related parameters were reported elsewhere.^11^ Shortly, the weight gain and food intake were similar between the groups. In addition, there was no statistically significant difference in the water intake and urine volume among the groups.

### 3.2 GX Diets Reduced Colon Crypt Depth

**Figure 1:**
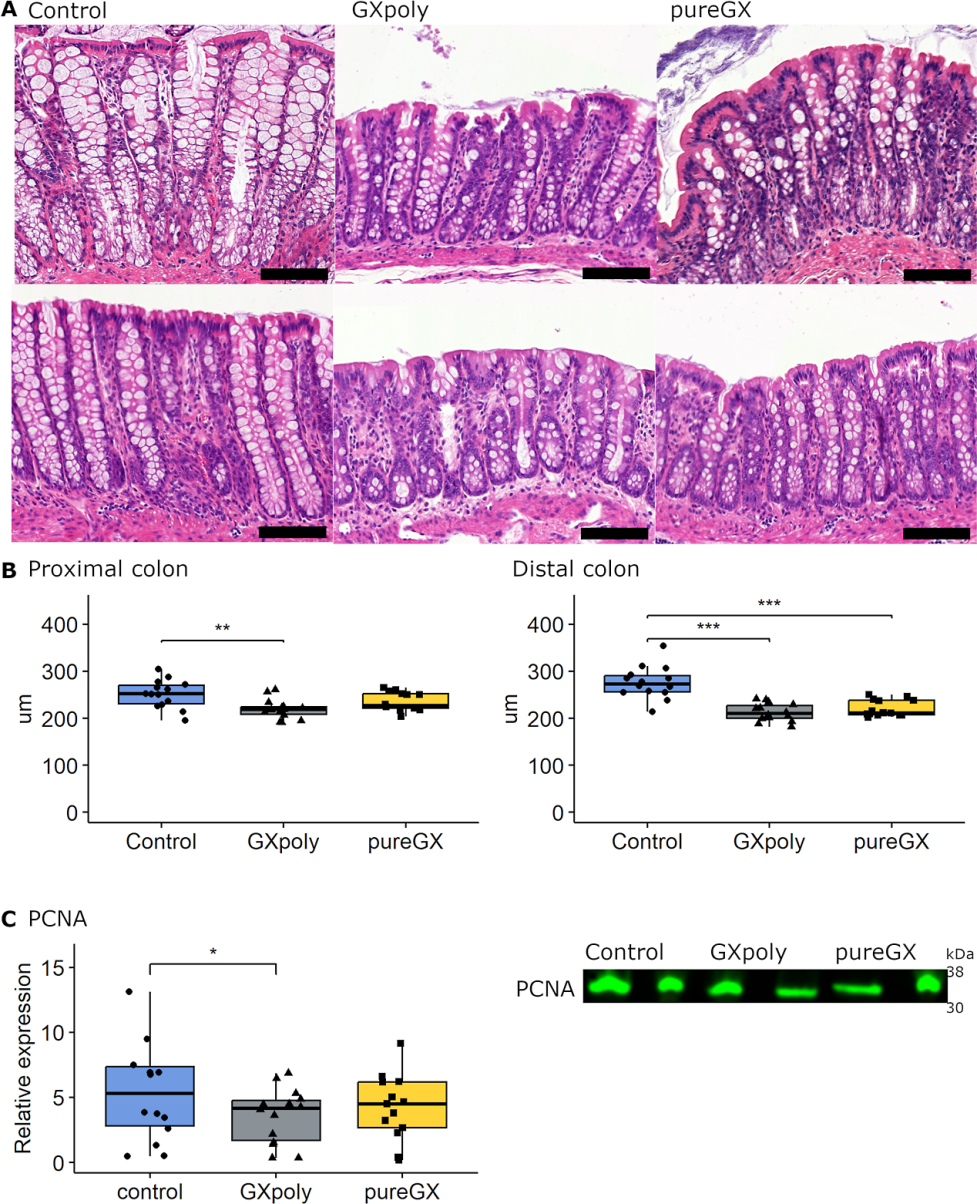
Colonic crypt depth and PCNA expression in healthy Wistar rats fed either control, GXpoly, or pureGX diet. (A) Representative hematoxylin and eosin histology in distal and proximal colons of rats fed control, GXpoly, or pureGX diets. Scale bar represents 100 μm. (B) Crypt depth in the proximal and the distal colon in the control, GXpoly, and pureGX groups after 28 days feeding period (*n* = 42). (C) PCNA relative expression levels, measured using western blotting, in the proximal colon of rats fed control, GXpoly, or pureGX diets (*n* = 41). To simplify the box and whiskers plot, one outlier from the control group (25.09) was removed. Boxes represent the median and interquartile range (IQR), with whiskers ±1.5× the IQR. Unprocessed western blot and total protein blot used for blot normalization are presented in Figure S1. (Kruskal–Wallis test for crypt depth, two-way ANOVA for PCNA, *p ≤ 0.05, **p ≤ 0.01, ***p ≤ 0.001, GXpoly = glucuronoxylan- and polyphenol-rich hemicellulose extract, pureGX = highly purified glucuronoxylan-rich hemicellulose extract, PCNA = proliferating cell nuclear antigen, PC = positive control).

Epithelial morphology was assessed, and the crypt depth was measured in longitudinal proximal and distal tissue samples employing H&E-stained sections (Figure 1A). In the distal colon, the crypts were lower in both GX groups than in the control group (*p* < 0.001) (Figure 1B), whereas in the proximal colon, the depth reduction was statistically significant only in the GXpoly group (*p* = 0.007). In line with the lower crypts, the expression of cell proliferation marker PCNA measured by Western blot was reduced in the proximal colon of rats fed the GXpoly diet in comparison to the control group (*p* = 0.048; Figure 1C). PCNA was not measured in the distal colon due to the lack of tissue material. The exact means ± SD of the results are reported in Table S2.

### 3.3 GX Diets Decreased the Absolute Number of Goblet Cells and Mucus Production

**Figure 2:**
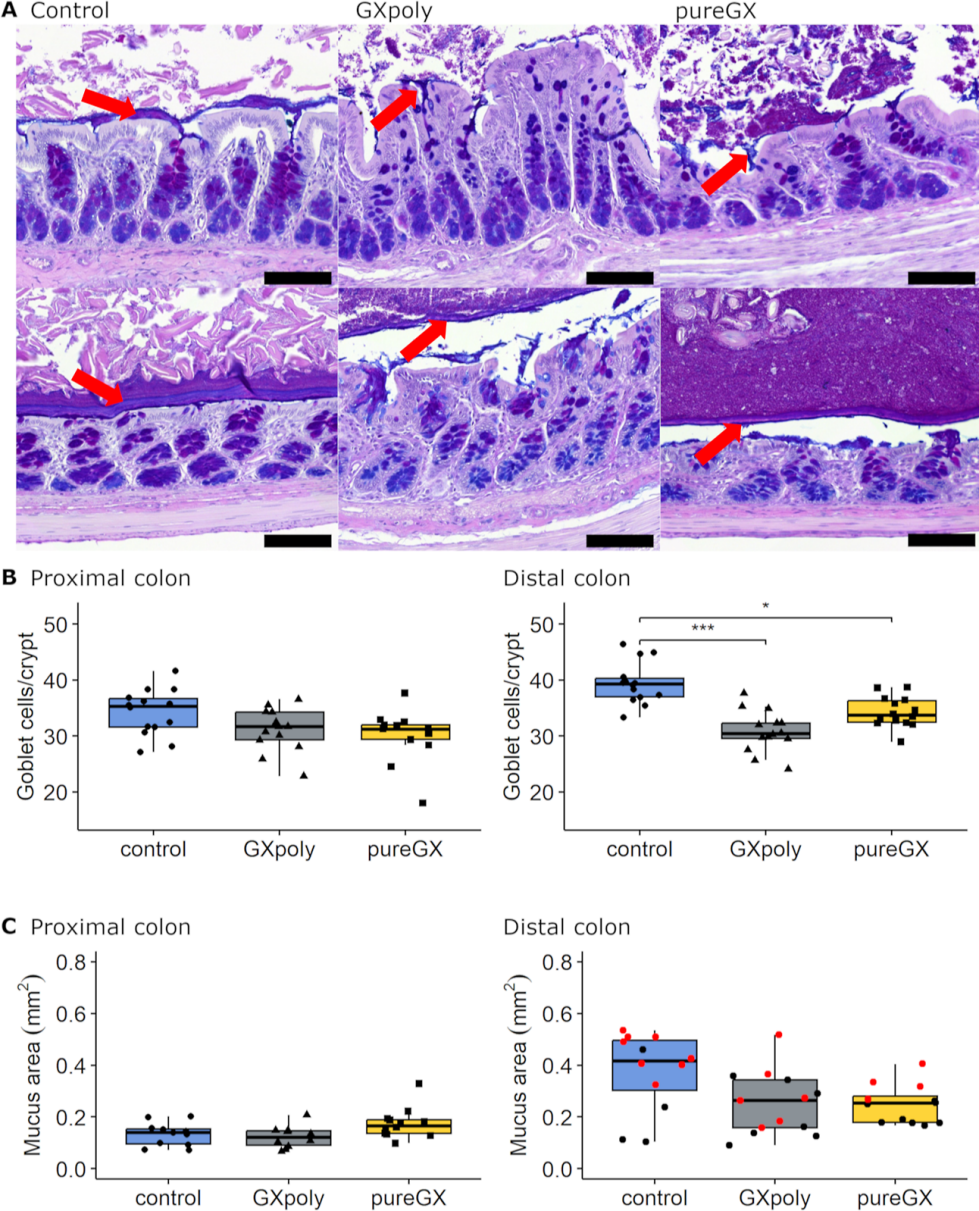
Mucus area (mm^2^) and number of goblet cells per crypt in the proximal and distal colon of rats fed either control, GXpoly, or pureGX diet. (A) Representative AB–PAS-stained image of the colon mucus layer (red arrows) in the proximal and distal colon. Scale bar represents 100 μm. (B) Goblet cell number per crypt in the proximal (*n* = 40) and the distal colon control (*n* = 42). (C) Colon mucus content in the proximal (*n* = 33) and the distal colon (*n* = 37). The distal colon samples without colonic content are marked with black dots and those with colonic content (*n* = 19) are marked with red dots in the box and whisker plot [boxes represent the median and interquartile range (IQR), with whiskers ±1.5× the IQR]. (Kruskal–Wallis test, *p ≤ 0.05, **p ≤ 0.01, ***p ≤ 0.001, GXpoly = glucuronoxylan- and polyphenol-rich hemicellulose extract, pureGX = highly purified glucuronoxylan-rich hemicellulose extract, AB = Alcian blue, PAS = periodic acid Schiff).

The AI-assisted investigation of AB–PAS-stained colon sections (Figure 2A and S2) showed that the number of goblet cells per crypt was higher in the control group than in the GX groups in the distal colon (*p* < 0.001), but not in the proximal colon (*p* = 0.061) (Figure 2B). However, the corresponding numbers of goblet cells divided by crypt depths showed no difference between the GX groups and the control group in distal or proximal colon (*p* = 0.074 and *p* = 0.405, respectively; Table S1). The AB/PAS cell ratio was not different between the groups either in the proximal (*p* = 0.147) or in the distal colon (*p* = 0.497). Consistent with the goblet cell results, the mucus area in the proximal colon was similar in all three dietary groups (*p* = 0.076), and it did not differ between the groups in the distal colon either (*p* = 0.056) (Figure 2C). On the other hand, the mucus area appeared to be dependent on the presence of the colonic content in the sample: When distal colon samples with and without colonic content were analyzed separately, the mucus area of samples with colonic content was higher in the control group than in the GXpoly group (*p* = 0.04), while the mucus area between the control and the pureGX group or between the GX groups did not show statistical difference. The dietary fiber source of the diet did not affect the mucus area in the empty colon (*p* = 0.354).

### 3.4 GX and Control Diets Had Similar Effects on Tight Junction Proteins

**Figure 3:**
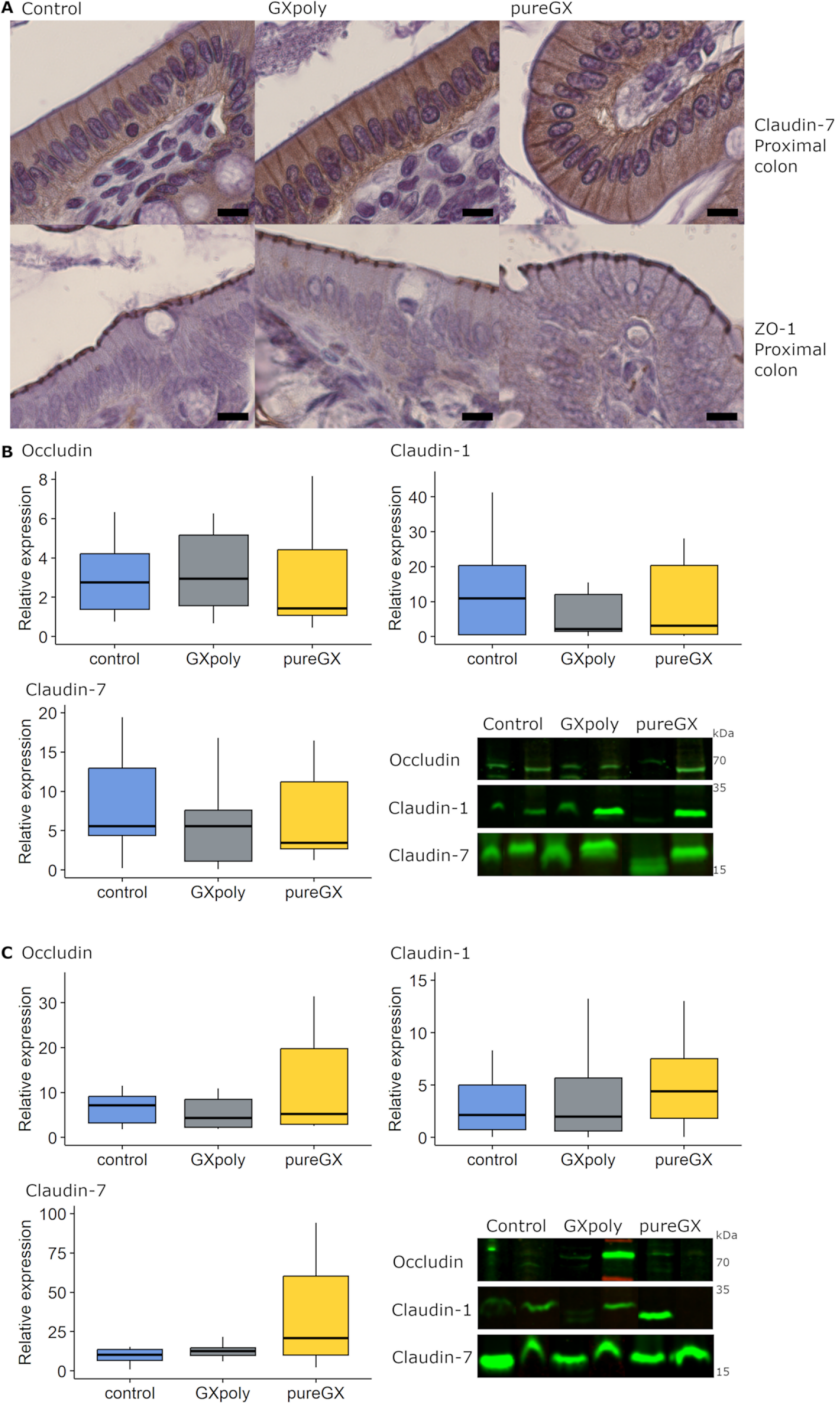
Tight junction expression in Wistar rats fed either control, GXpoly, or pureGX diet. (A) Representative images of claudin-7 and ZO-1 expression in proximal colons. Scale bar represents 10 μm. (B) Box and whiskers plots [boxes represent the median and interquartile range (IQR), with whiskers ±1.5× the IQR] display the relative expression of measured tight junction proteins in the proximal colon (*n* = 41). Outliers are not presented in the plots to make them easier to interpret [occludin: outliers of the control group (27.5, 31.0) and the pureGX group (11.4) not shown; claudin-7: outliers of the control group (34.1, 52.7) and the GXpoly group (22.6) not shown]. (C) Relative expression of occludin [outliers of the control group (52.3) and the pureGX group (93.5) not shown], claudin-1 [outliers of the control group (31.6) and the pureGX group (90.9) not shown], and claudin-7 [outlier of the control group (81.8) not shown] in the distal colon (*n* = 39). Unprocessed western blots and total protein blots used for blot normalization are presented in Figure S1. (two-way ANOVA, *p ≤ 0.05, **p ≤ 0.01, ***p ≤ 0.001, GXpoly = glucuronoxylan- and polyphenol-rich hemicellulose extract, pureGX = highly purified glucuronoxylan-rich hemicellulose extract, ZO-1 = zonula occludens-1).

Localization of proteins claudin-7 and ZO-1 was confirmed using immunohistochemistry (Figure 3A). To compare the potential differences in mucosal permeability between the groups, the expression of occludin, claudin-1, and claudin-7 was analyzed by western blotting (Figure 3B,C). In the proximal colon, differences in the relative expression of the tight junction proteins occludin (*p* = 0.335), claudin-1 (*p* = 0.514), and claudin-7 (*p* = 0.078) between the groups were not statistically significant. Similarly, in the distal colon, there was no statistically significant difference in the expression of tight junction proteins (occludin *p* = 0.177, claudin-1 *p* = 0.701, claudin-7 *p* = 0.164). However, even though statistical significance was not achieved due to high deviation, a trend toward higher expression of, especially, occludin and claudin-7 could be seen in the pureGX group (Figure 3C). In the proximal colon, the relative expression of claudin-1 was higher in females than in males (*p* < 0.001).

**Figure 4:**
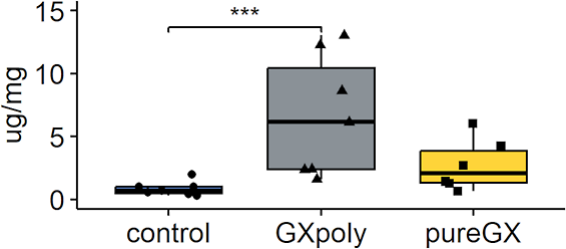
Fecal IgA concentration in healthy Wistar rats fed either control, GXpoly, or pureGX diet. Fecal IgA concentration measured from the proximal colon samples (*n* = 20). Boxes of the plot represent the median and interquartile range (IQR), with whiskers ±1.5× the IQR. (two-way ANOVA, *p ≤ 0.05, **p ≤ 0.01, ***p ≤ 0.001, GXpoly = glucuronoxylan- and polyphenol-rich hemicellulose extract, pureGX = highly purified glucuronoxylan-rich hemicellulose extract, IgA = Immunoglobulin A).

To further assess the intestinal permeability, we measured the serum LBP concentration. As serum LBP levels were under the detection limit, we were not able to measure the difference between the groups in the LBP concentration. To estimate the differences in immune function between the groups, we measured the fecal IgA concentration. IgA was higher in the rats fed GXpoly diet that in the control group (Figure 4). However, there was no difference between pureGX and the control group.

### 3.5 Colon Gene Expression Profiles and Differently Modulated Genes between the GX Groups and the Control Group

**Figure 5:**
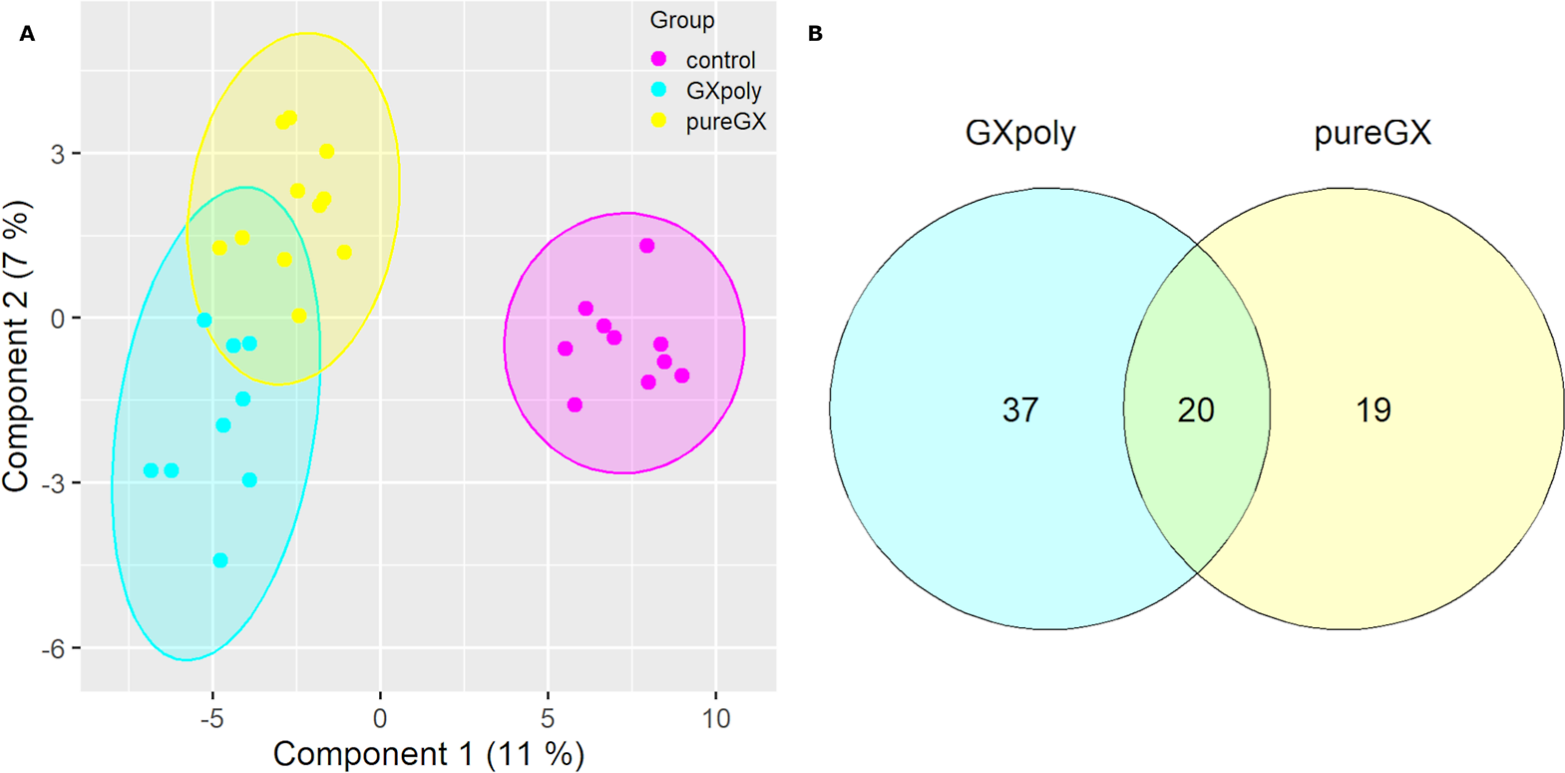
Effects of GXpoly and pureGX on gene expression profiles in colon epithelium. (A) PLS-DA score plot for comparison of effects of the GXpoly, pureGX, and cellulose diets on gene expression profiles in proximal colon. Colored dots inside the data ellipses represent individual samples. (B) Differently expressed genes in the proximal colon tissue of rats fed either the pureGX or GXpoly diet when compared to the control diet (*n* = 30). Genes in common between GX groups are on the middle of the diagram. Upregulated genes marked in green and downregulated genes are marked in red. Used cutoff values were |FC| ≥ 1.5 and adj. *p*-value ≥ 0.05. (PLS-DA = partial least-squares-discriminant analysis, GXpoly = glucuronoxylan- and polyphenol-rich hemicellulose extract, pureGX = highly purified glucuronoxylan-rich hemicellulose extract).

Expression profiling by microarray was used to assess the impact of GX-rich diets on genome-wide gene expression in the colon epithelium. Individual rats were compared based on their gene expression profile and diet using sPLS-DA. Colon gene expression profiles showed some clustering, which indicated differences in gene expression profiles between the GX groups and the control group (Figure 5A). Between GXpoly and pureGX, separation was less distinct. When individual gene expressions were compared, 39 genes in the pureGX group and 57 in the GXpoly group showed at least 1.5-fold difference in expression when compared to that observed in the control group (adj. *p*-value ≥ 0.05) (Figure 5B). Of these differently expressed genes, 20 were common between the pureGX and GXpoly groups.

On restricting changes to genes showing a twofold difference, 6 genes in GXpoly and 6 genes in pureGX were upregulated and 15 genes in GXpoly and 7 genes in pureGX were downregulated when compared to the control group. The two genes with the highest upregulation in the both GX diets were actin binding- and deoxyribonuclease activity-related *Dnase1* and calcium binding-related *S100g* (Table 1). Other upregulated genes in both GX groups were *Aqp8* (water transporter), *Cyp3a18* (cytochrome P450 3a18), and *Cmahp* (cytidine monophospho-*N*-acetylneuraminic acid hydroxylase pseudogene),which were some of the most upregulated genes in the GX groups. In addition, the *Slc22a3* (organic cation transporter) gene was upregulated in the GXpoly and *Ly6d* (lymphocyte antigen 6 family member D) in the pureGX group when compared to the control group.

**Table 1:** Fold Changes of Differentially Expressed Up- and Downregulated Genes by ≥ Twofold in the GXpoly and PureGX Groups When Compared to the Control Group^a^

symbol	GXpoly FC	adj. *p*-value	pureGX FC	adj. *p*-value
Dnase1	8.393	0.0035	6.133	0.0114
S100g	4.295	0.0022	3.675	0.0059
Slc22a3	2.479	0.0004	*1.986*	*0.0049*
Aqp8	2.337	0.0087	2.475	0.0059
Cyp3a18	2.188	0.0022	2.016	0.0059
Cmahp	2.075	0.0087	2.266	0.0049
Ly6d	1.155	0.7627	2.291	0.0317
Fos	0.480	0.0476	*0.486*	*0.0540*
Arhgef3	0.473	0.0062	*0.719*	*0.1754*
LOC102555398	0.464	0.0036	0.485	0.0062
Anxa1	0.463	0.0063	*0.518*	*0.0168*
Rtp4	0.458	0.0126	*0.652*	*0.1357*
LOC108348254	0.440	0.0147	*0.577*	*0.0348*
Samd9	0.437	0.0063	*0.543*	*0.0063*
LOC100912658	0.436	0.0274	*0.534*	*0.0816*
LOC102548270	0.433	0.0147	0.469	0.0276
Tppp3	0.418	0.0436	*0.537*	*0.1309*
Svep1	0.417	0.0126	*0.932*	*0.0643*
Apol9a	0.403	0.0022	*0.579*	*0.0541*
Ly6m	0.402	0.0094	0.495	0.0421
Wfdc1	0.392	0.0115	0.419	0.0174
Rsad2	0.342	0.0438	*0.353*	*0.0525*

a Values not statistically significant or not ≥twofold up- or downregulated are presented in italics.

One of the most downregulated genes in pureGX and the GXpoly group was *Wfdc1* (WAP Four-Disulfide Core Domain 1). Other genes downregulated in both groups were *Rsad2* (codes an interferon-inducible antiviral protein), *Ly6m* (lymphocyte antigen 6 complex, locus M), *Anxa1* (calcium binding), and *Fos* (Fos proto-oncogene, AP-1 transcription factor subunit). The GXpoly group had more downregulated genes than pureGX when compared to the control group. Genes downregulated only in the GXpoly group were *Apol9a* (lipid transport and lipoprotein metabolic processes), *Svep1* (Sushi, Von Willebrand Factor Type A, EGF And Pentraxin Domain Containing 1), *Tppp3* (tubulin polymerization), *Samd9* (Sterile Alpha Motif Domain Containing 9), *Rtp4* (Receptor Transporter Protein 4), and *Arhgef3* (autophagy).

## 4 Discussion

The birch-derived fiber extracts GXpoly and pureGX have multifunctional properties as food stabilizers, and they are also a potential source of the dietary fiber GX. Furthermore, GXpoly is rich in polyphenolic lignin, whereas pureGX contains only a scarce amount of lignin and other polyphenols. We have previously reported that when compared to cellulose, GXpoly and pureGX diets resulted in different gut microbiota and their metabolites in healthy Wistar rats.^11^ It was unknown whether such effects of GX-rich extracts on the colonic microbiome result in differences in the colon mucosal physiology. In the present study, various biomarkers of colonic physiology and intestinal barrier function were analyzed, which allowed an in-depth investigation of potential mechanisms and differences between the three diets. We used a modified AIN-93G diet as a control, which differed from the basal AIN-93G only in the concentration of cellulose, set at 10% (w/w). In comparison to the control diet, both GX-rich diets resulted in lower crypts particularly in the distal colon, which was accompanied by reduced expression of the cell proliferation marker PCNA. However, only mild changes could be observed in the expression of tight junction proteins, number of goblet cells, and mucus production assessed as mucus area. Interestingly, the GXpoly diet induced a markedly higher luminal IgA concentration compared with that of the cellulose-containing diet.

Both GX-rich diets resulted in a reduction of crypt depth in comparison to the cellulose diet, the effect being more pronounced in the distal colon and by the GXpoly diet. This result was accompanied by a lower expression of the proliferation marker PCNA, indicating that the differences in crypt depth were likely explained by lower cell proliferation in the rats fed GX diets. This result is in line with previous animal studies showing that midcolon crypt depth was lower in pigs fed arabinoxylan than in pigs fed cellulose, although the difference was not statistically significant,^2^ and that high cellulose supplementation increased epithelial cell proliferation in a mouse model of endotoxemia.^21^ In contrast, barley arabinoxylan did not affect cell proliferation in intestinal epithelial cells in vitro.^22^ Furthermore, djulis, which is a polyphenol-rich cereal food, decreased cell proliferation in a rat model of colorectal cancer.^23^ Difference in crypt depth in our study may be related to changes in the gene expression, as GX exhibited stimulation of two genes associated with apoptosis (*S100g* and *DNase1*) and decreased the expression of a gene associated with cell proliferation (*Wfdc1*). Highly fermentable fructo-oligosaccharides have been associated with the upregulation of apoptosis-related genes and downregulation of genes involved in cellular proliferation in the proximal colon compared to wood cellulose in healthy rats,^24^ and different types of fibers can modulate colon gene expression in a similar way, partly explained by the similarity in their fermentation profiles.^25^ The higher content of fermentable material in the GX-rich diets than in the control diet may partly explain the difference in gene expression in the midcolon and, as a result, the difference in crypt depth.

The more distinct effect of GXpoly on the crypt depth and cell proliferation is most likely explained by the high lignin content in the diet. We have shown that lignin is negatively associated with a cecal abundance of *Akkermansia*,^26^ which was lower in the GXpoly group than in the control group presented in our previous research.^11^ In a recent study, supplementation with *A. muciniphila* was associated with increased cell proliferation in a mouse model of colorectal cancer.^27^ Various polyphenols such as lignans, quercetin, and curcumin have been shown to be able to inhibit the expression of genes and proteins related to cell proliferation and induce apoptosis in vitro and in vivo.^28,29^ Concomitantly, mRNA expression of a known oncogene, *Fos*, was lower in the GXpoly group than in the control group, while its expression did not show a statistical difference between pureGX and the control group. In general, epithelial cell turnover in the colon is high, allowing for the removal of potentially infected or damaged cells,^30^ but the balance between cell apoptosis and proliferation is crucial as hyperproliferation is associated with DNA damage and, therefore, increased cancer risk.^31^ Further studies using a colorectal cancer model are needed to elaborate the mechanisms of GX-rich extracts as a potential antiproliferative agent.

In addition to *Fos*, some other genes associated with cancer, such as *Anxa1*, *Samd9*, *Tppp3*, *Slc22a3*, and *Ly6d*, were differently regulated in both or one of the GX-rich groups, compared with the control group. *Slc22a3* variants have been associated with colorectal cancer in the Asian population,^32^*Samd9* was expressed in lower levels in colon cancer tissue than in normal colon,^33^*Tppp3* knockdown inhibited cell proliferation in colon cancer cell lines in vitro,^34^*Ly6d* was expressed in high levels in colorectal cell lines relative to normal fibroblasts,^35^ and ANXA1 increased the stability of tumor growth increasing EPH receptor A2 in human colon cancer cells in vitro.^36^ Generally, foods containing dietary fiber have been associated with a reduced risk of colorectal cancer;^37^ however, some of the differences in the expression of cancer-associated genes in this study indicate that cellulose, more than GX, has the potential to reduce colorectal cancer risk and vice versa. However, the relationship between the mRNA regulation of these genes and susceptibility to colorectal cancer has not been well-defined.

The mucus layer is a compact, viscous, and permeable gel working as a shield between intestinal epithelia and environmental hazards.^38^ It has been shown that in the distal colon, a firm mucus layer covers the fecal pellet, while in the proximal colon, close contact between bacteria and epithelia can be observed as there is no intact mucus layer.^16^ The backbone of the mucus layer is composed of mucin proteins synthesized by intestinal goblet cells.^38^ To our knowledge, this is the first study in which AI has been utilized to measure colonic mucus, enabling the comparison of the whole mucus layer area of the transversal colon samples. In the proximal colon, there was no difference in the mucus content or the number of mucus-producing goblet cells between the groups. This result is in line with a previous study where cecal digesta mucin content was the same in rats fed either xylo-oligosaccharide- or cellulose-containing diet.^39^ As presence of fecal material has shown to affect the mucus layer thickness in distal colon,^16^ we measured the distal colon samples with and without colonic content separately. There the cellulose seemed to work better at inducing mucus production than GX as both the mucus area of the samples with colonic content and the number of goblet cells were higher in the control group than in the GX-rich groups. The difference in the number of goblet cells cannot be explained by the increased differentiation of colon transit-amplifying cells into goblet cells, as the goblet cell-to-enterocyte ratio measured as goblet cells per crypt depths was the same in every group. Instead, the higher cell proliferation in the control group than in the GX groups may induce the difference.

The different results between the proximal and distal colon mucus and goblet cell amounts may result from differing microbial metabolite, such as SCFA, concentrations between these two sites. As microbial metabolites are generally rapidly utilized by bacteria or absorbed by colon epithelial cells,^1^ the SCFA concentration decreases along the colon.^40^ In our previous study, we showed that when compared to cellulose, GX diets led to higher cecal bifidobacteria and SCFA concentration (Table S3),^11^ which have previously been positively associated with mucus layer thickness and mucus production.^38^ As there was no statistically significant difference in distal colon SCFA content between the groups,^11^ the stimulation of mucus production by SCFA may not have been as efficient in the distal colon as in the proximal colon. A higher abundance of *Akkermansia* in the control group than in the GX groups may partially explain the difference in the mucus layer area between the groups as *A. muciniphila* has been associated with increased mucus layer thickness.^38^ Our study suggests that mechanical friction caused by insoluble cellulose seems to be at least as effective at inducing mucus production as alterations in gut microbiota and colonic metabolism caused by GX.

Epithelial cells and intercellular junctions form the intestinal physical barrier.^4^ Its function depends on the paracellular barrier, which is maintained by tight junctions among intestinal epithelial cells.^4^ Tight junction proteins such as occludins, claudins, zonula occludens (ZOs), and junction adhesion molecules (Jams) regulate the paracellular transport of ions and small water-soluble solutes.^1^ They are connected with epithelial cells and with each other; transmembrane proteins such as occludins and claudins are anchored to the cytoskeleton through cytosolic proteins, including ZOs.^41^ Disturbance of tight junction proteins may increase intestinal permeability and lead to bacterial translocation, which induces inflammation and can contribute to several intestinal diseases.^42^ We did not notice a difference in occludin, claudin-1, or claudin-7 expression between the groups in the proximal or distal colon. This result is in line with a previous study, where insoluble fiber supplementation did not affect occludin or claudin-3, -4, or -7 expressions in healthy mice.^43^ In addition, the localization of claudin-7 and ZO-1 was the same in all of the groups based on immunohistochemistry.

Previously, polyphenols have shown to improve the colonic TJ barrier in humans with increased intestinal permeability^44^ and in a colitis mouse model.^45^ Birch wood-derived extract rich in polyphenols did not have a distinct effect on tight junction proteins compared to cellulose or highly purified GX in healthy rats. Polyphenols make up a heterogeneous group of compounds and may affect healthy and diseased individuals differently. Thus, to further decipher the potential of lignin and lignin-derived compounds on intestinal permeability, they should also be studied in diseased models.

Previous studies in humans and mice have found sex-based differences in tight junction protein expression.^46,47^ We found that the relative expression of claudin-1 in the proximal colon was higher in female rats than in male rats; however, the individual variation in the tight junction protein expression was high. Even though sex seems to be associated with the expression of tight junction proteins, studies addressing the differences in their expression are scarce. Thus, more studies are needed to address the mechanisms of sex hormones on intestinal permeability, including tight junction protein expression. Furthermore, sex should be considered as a cofounder in studies measuring intestinal permeability or tight junction protein expression. No differences were observed in any other measured variables between the male and female rats.

Secretory IgA, produced by plasma cells in the lamina propria, is a part of the immunological barrier that reinforces protective intestinal barrier function by limiting the opportunistic invasion of pathogenic microbes.^1^ Depletion of IgA can lead to an increased innate immune response.^30^ Fecal IgA content was higher in the GX-rich groups than in the control group. However, the larger, statistically significant increase, only in the GXpoly group, suggests that birch polyphenols and GX are together better at increasing IgA secretion than GX alone. Polyphenols from different sources can have different effects on IgA production, as berry polyphenols were able to increase,^48^ and cocoa polyphenol-rich diets decrease^49^ the fecal IgA production in healthy rats. Dietary supplementation with wheat bran, which naturally contains lignin, enhanced IgA production in mice.^7^ In addition, arabinoxylan- and the ferulic acid-rich extract was better at improving the overall immune function than the more purified arabinoxylan-rich extract.^50^ We have shown that lignin in GXpoly can be metabolized into ferulic acid.^26^ Thus, ferulic acid may be at least partly responsible for the higher IgA secretion stimulation in the GXpoly group. Overall, the results indicate that wood-derived polyphenols may stimulate IgA production, especially in the interaction with fermentable fibers.

This study demonstrates that despite the similarity in the gut microbiota and colonic metabolism profiles between the GXpoly and pureGX groups, modest differences between the extracts can further promote slightly different colonic functions in the healthy unchallenged state. GXpoly caused pronounced and more numerous changes in the intestinal barrier biomarkers than pureGX compared to the control group. This result indicates that fermentation alone does not explain the differences between the dietary fibers as lignin and cellulose are considered less fermentable than GX. GXpoly is rich in lignin and lignin-derived metabolites. Since the whole extracts were used in the study, it is impossible to determine whether lignin or lignin-derived components alone or combined effects of lignin and GX were responsible for the beneficial effects of GXpoly on the colonic immunological barrier or cell proliferation homeostasis. Generally, very little is known about the effects of lignin on colon health. Therefore, future studies to determine the specific impact of birch lignin and lignin-derived bioactive components on intestinal barrier function are needed.

In conclusion, the present study confirmed that different dietary fibers improve the colonic barrier function via distinct mechanisms. The control diet was better at inducing mucus production in the distal colon, but the GX-containing diets suppressed cell proliferation and, especially, the GXpoly diet supported the mucosal immune function in the colon. Diets containing GX-rich extracts regulated the colonic permeability and mucus area in the proximal colon as well as the cellulose-containing diet, which is considered to be healthy for rats. These findings provide evidence that GX-rich extracts have the potential to support the colonic barrier function as a future food ingredient.

## Supporting Information Available

Composition of the experimental diets and AIN-93G, intestinal barrier biomarkers by dietary group and sex of the rats, and short-chain fatty acid content of fecal and cecal samples (XLSX)
Original western blots and blots used for normalization of western blots, and representative image of AB–PAS-stained colon tissue samples (PDF)
