# Supplementary material for: Polyphenol- and Glucuronoxylan-Rich Fiber Extract from Birch (Betula sp.) Wood Regulates Colonic Barrier Function and Cell Proliferation in Healthy Rats
Source: J Agric Food Chem. 2024 Feb 12;72(7):3495–505. doi: 10.1021/acs.jafc.3c07757 (PMC11398711; doi:10.1021/acs.jafc.3c07757)
Supplement: Supplementary file 2 — jf3c07757_si_002.pdf [file jf3c07757_si_002.pdf]

Supporting information

Polyphenol- and Glucuronoxylan-Rich Fiber 0045xtract from Birch (*Betula* sp.) Wood Regulates Colonic Barrier Function and Cell Proliferation in Healthy Rats

Emma Kynkäänniemi[a*], Jere Lindén[b], Suchaya Ngambundit[a], Laura A. Saarimäki[c], Dario Greco[cd], Hana Slaba[a], Maarit H. Lahtinen[a], Kirsi S. Mikkonen[ae], Anne-Maria Pajari[a*]

[a] Department of Food and Nutrition, University of Helsinki, 00014 Helsinki, Finland

[b] Department of Veterinary Biosciences, and Finnish Centre for Laboratory Animal Pathology (FCLAP), Helsinki Institute of Life Science (HiLIFE), University of Helsinki, 00014 Helsinki, Finland

[c] Finnish Hub for Development and Validation of Integrated Approaches (FHAIVE), Faculty of Medicine and Health Technology, Tampere University, 33520 Tampere, Finland

[d] Division of Pharmaceutical Biosciences, Faculty of Pharmacy, University of Helsinki, 00014 Helsinki, Finland

[e] Helsinki Institute of Sustainability Science (HELSUS), P.O. Box 65, 00014, University of Helsinki, Finland.

* Correspondence to Emma Kynkäänniemi, +358504760649, emma.kynkaanniemi@helsinki.fi[*]

Supplementary figures.

PCNA

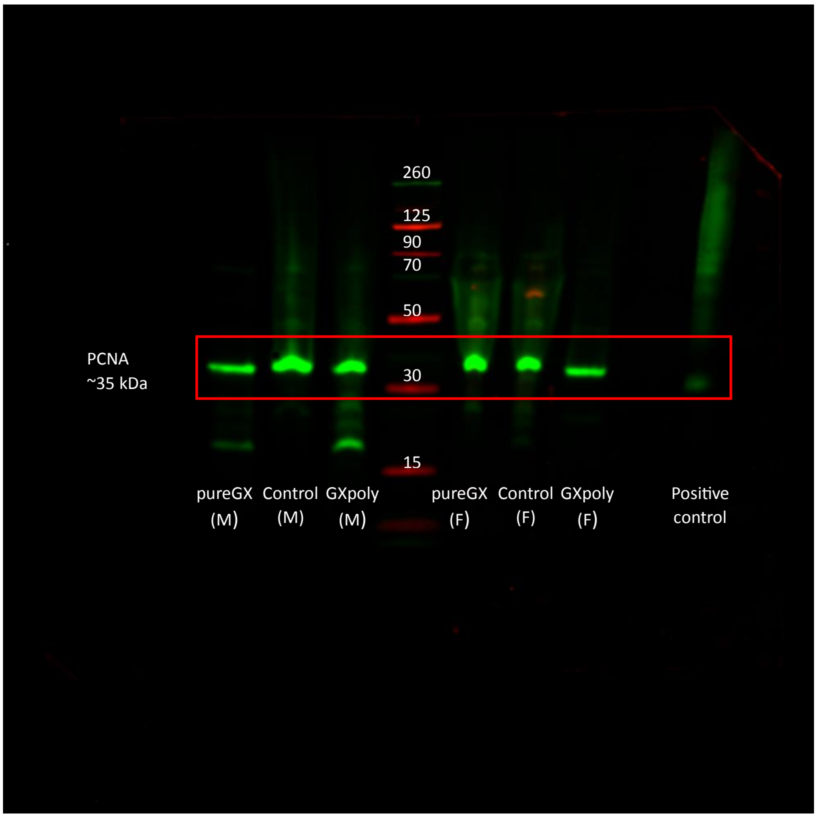

Total protein

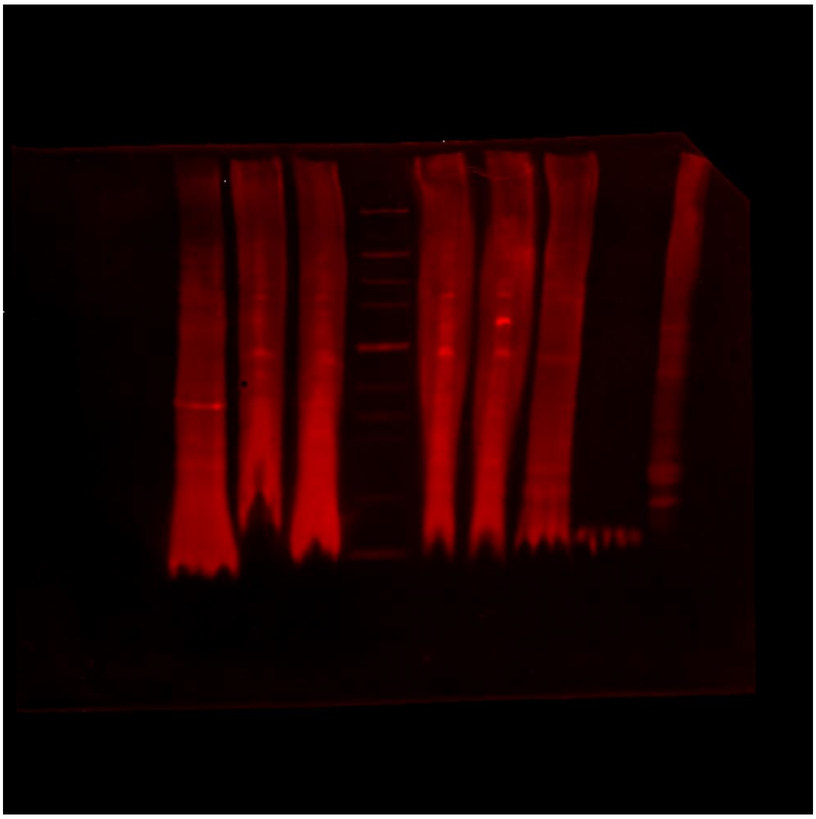

Occludin proximal colon

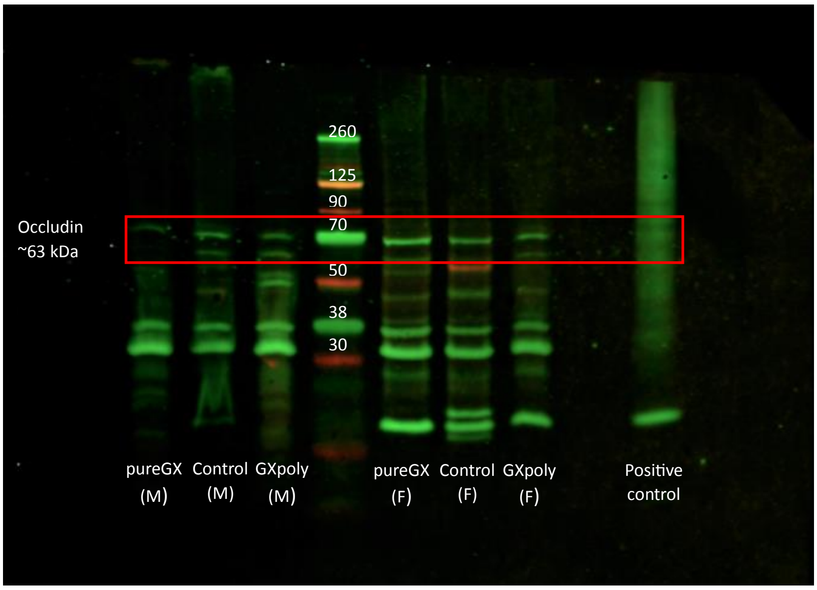

Total protein

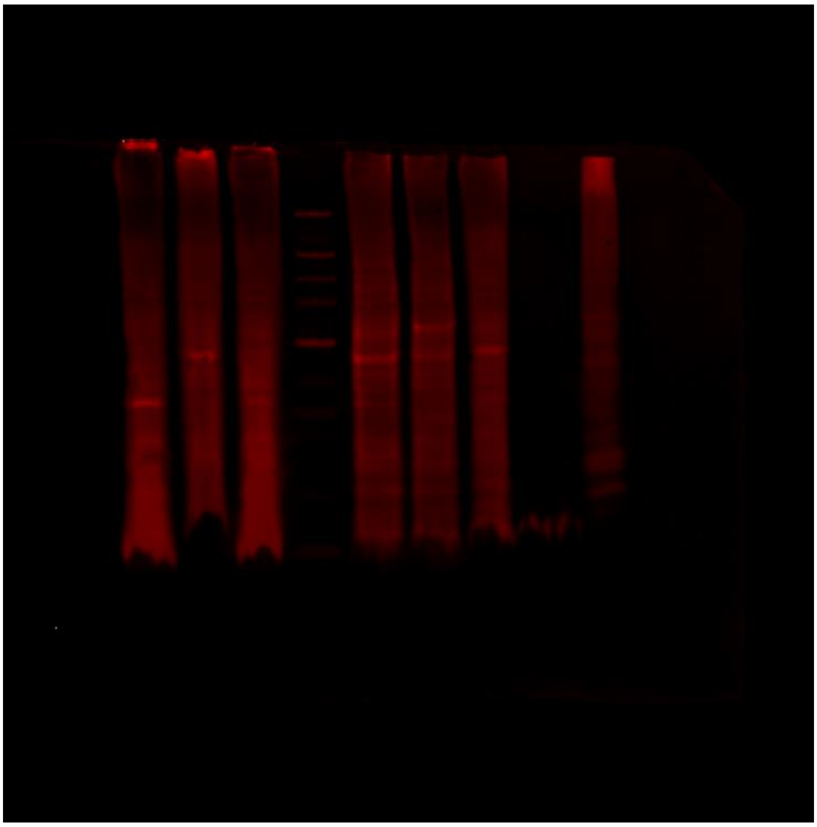

Claudin-1 proximal colon

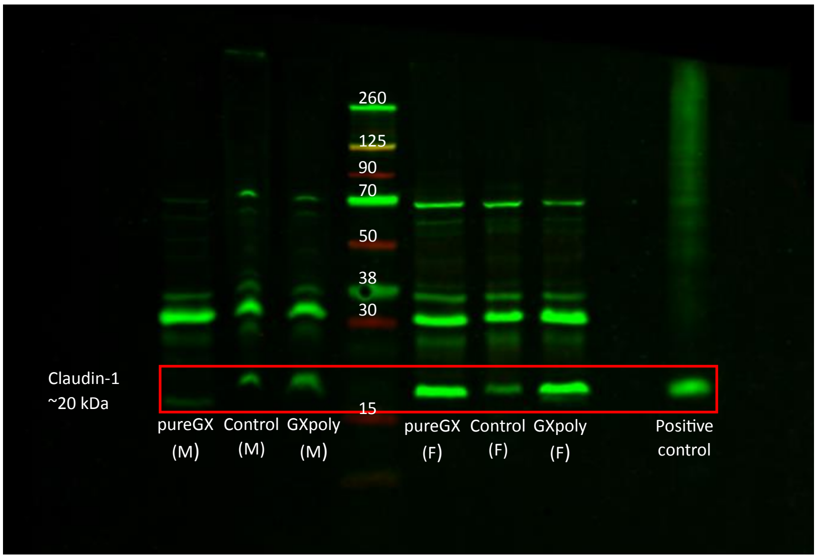

Total protein

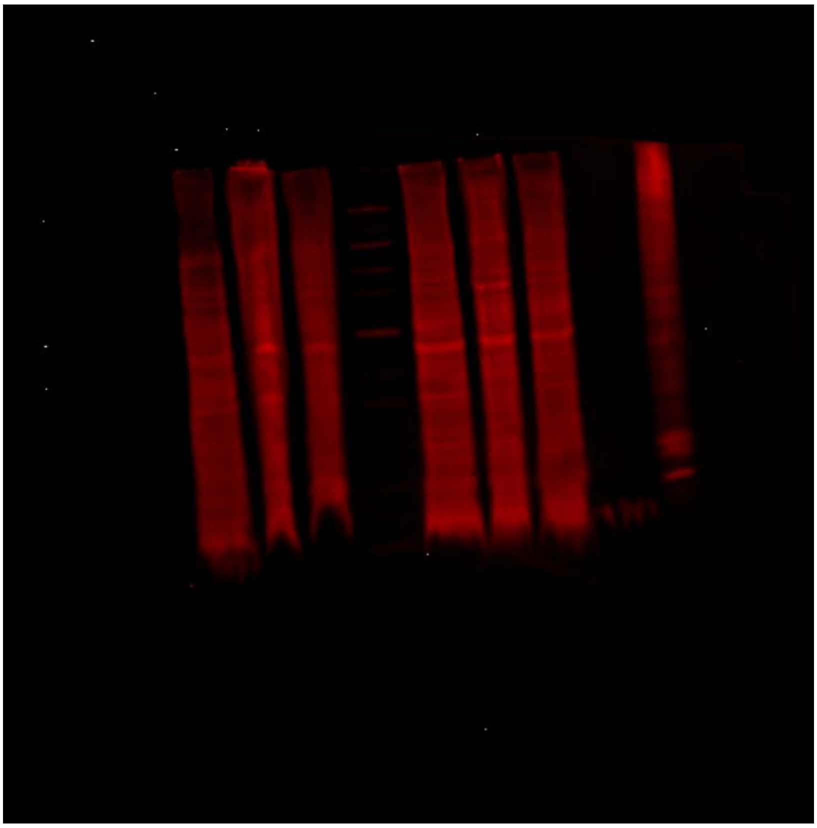

Claudin-7 proximal colon

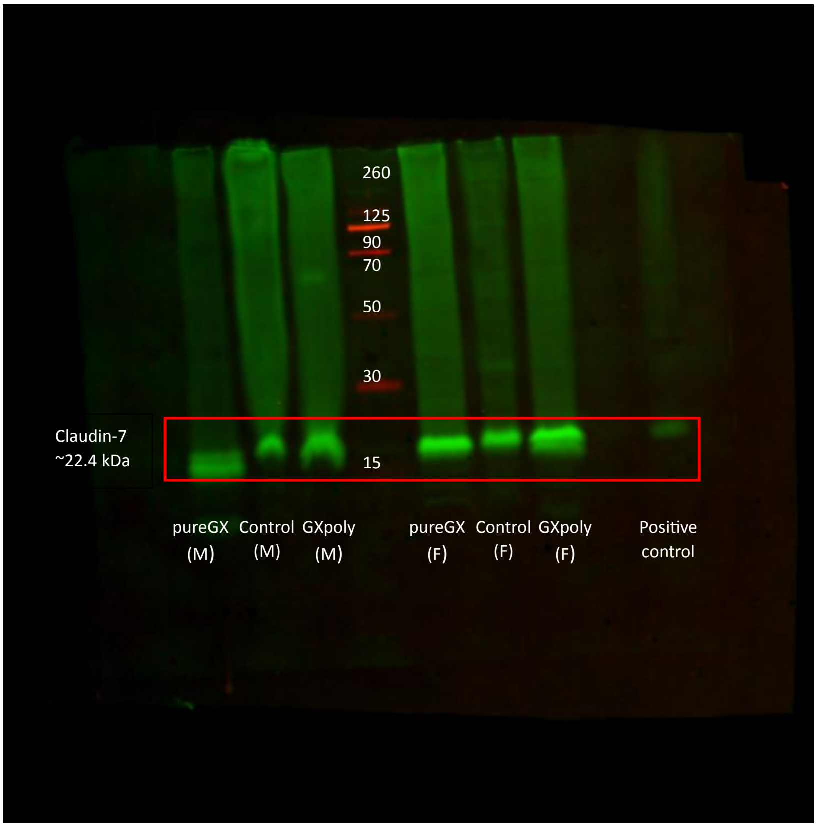

Total protein

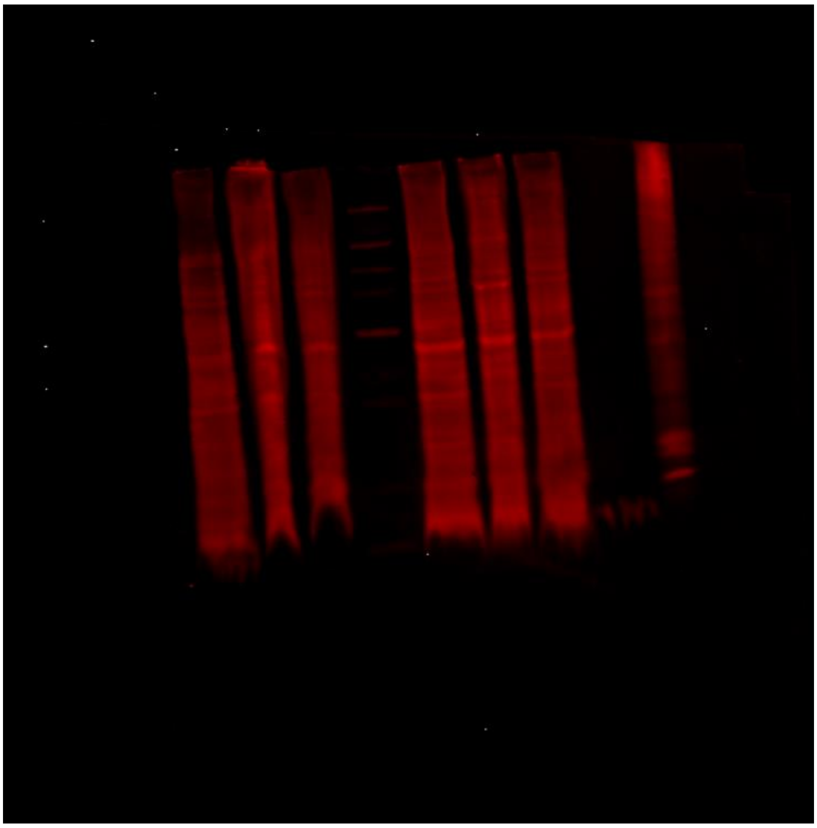

Occludin distal colon

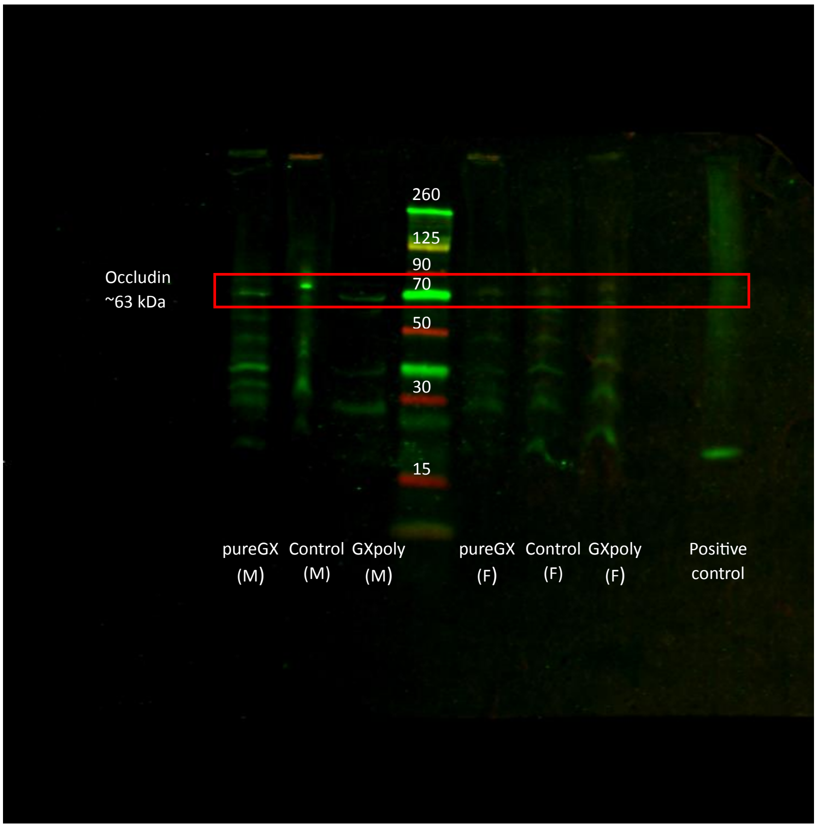

Total protein

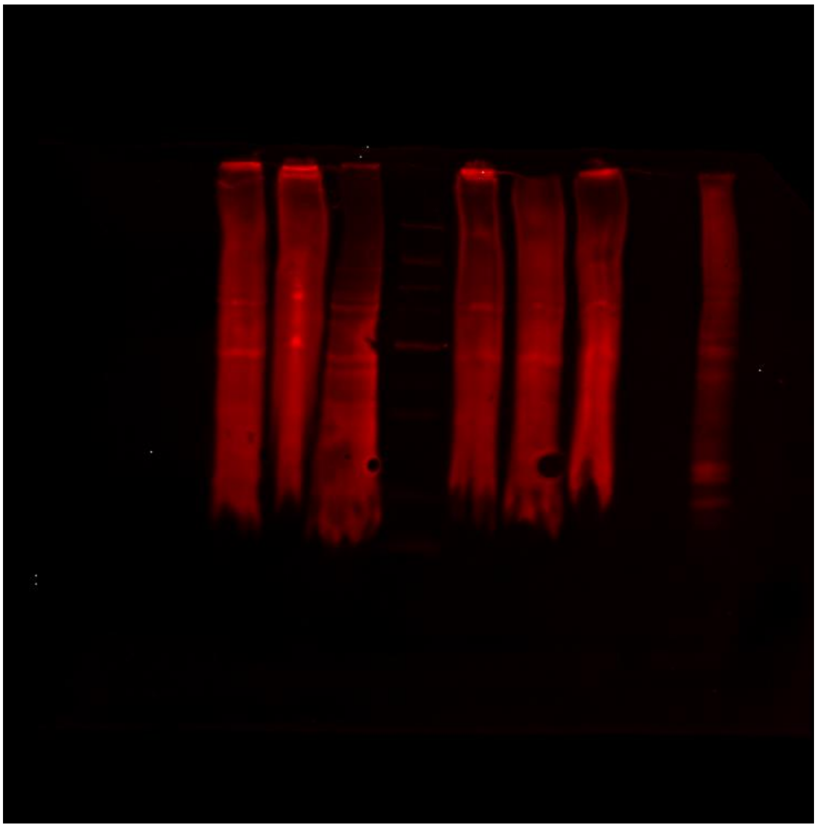

Claudin-1 distal colon

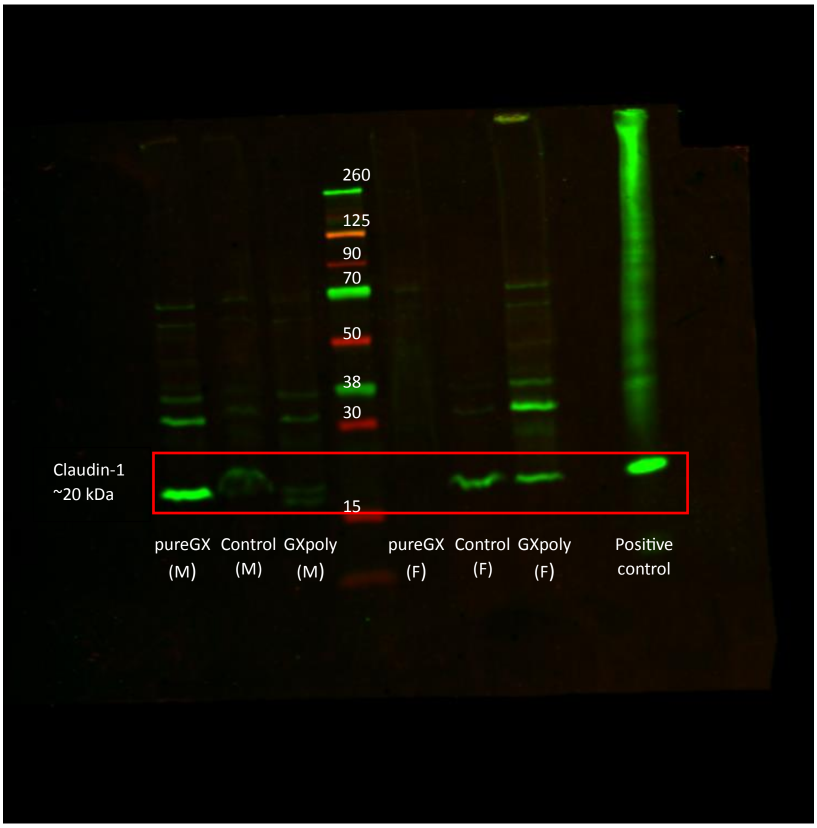

Total protein

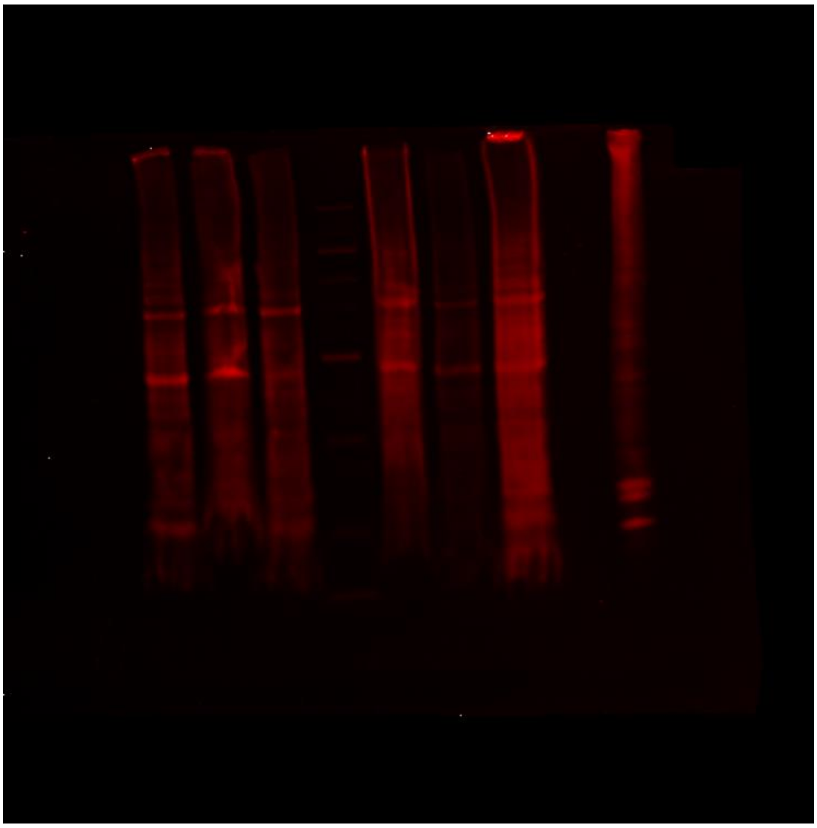

Claudin-7 distal colon

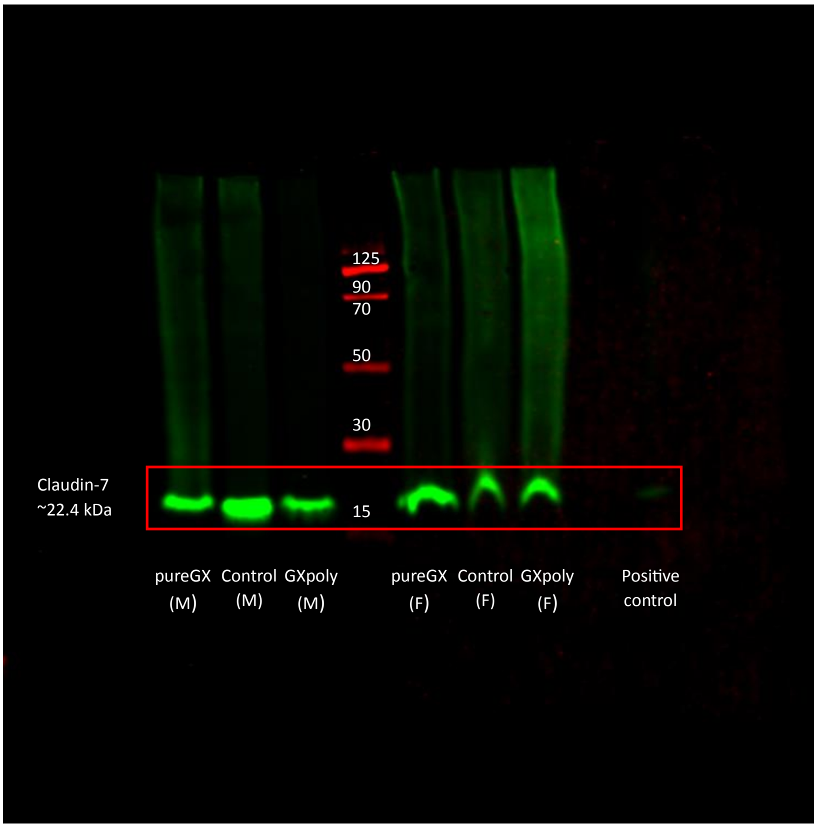

Total protein

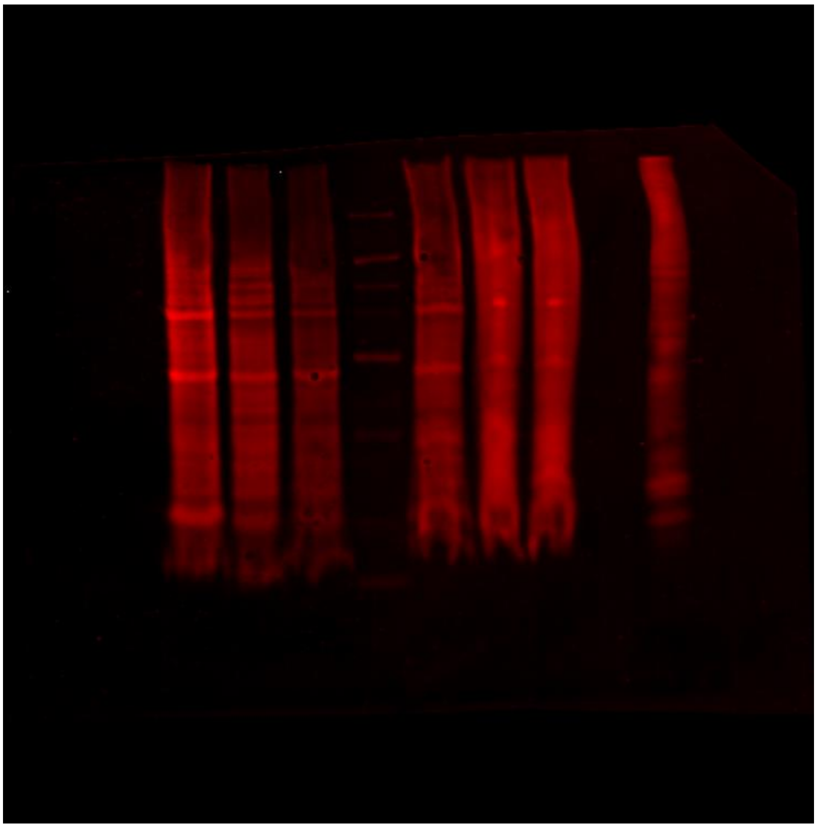

Figure S1. Original Western blots and total protein blots used in their normalization. The uncropped, untouched, full original image of western blots presenting the proximal colon PCNA, and proximal and distal colon occludin, claudin-1 and claudin-7 of the rats fed either GXpoly, pureGX or cellulose containing diet (PCNA = proliferating cell nuclear antigen, GXpoly =

glucuronoxylan- and polyphenol-rich hemicellulose extract, pureGX = highly purified glucuronoxylan-rich hemicellulose extract)

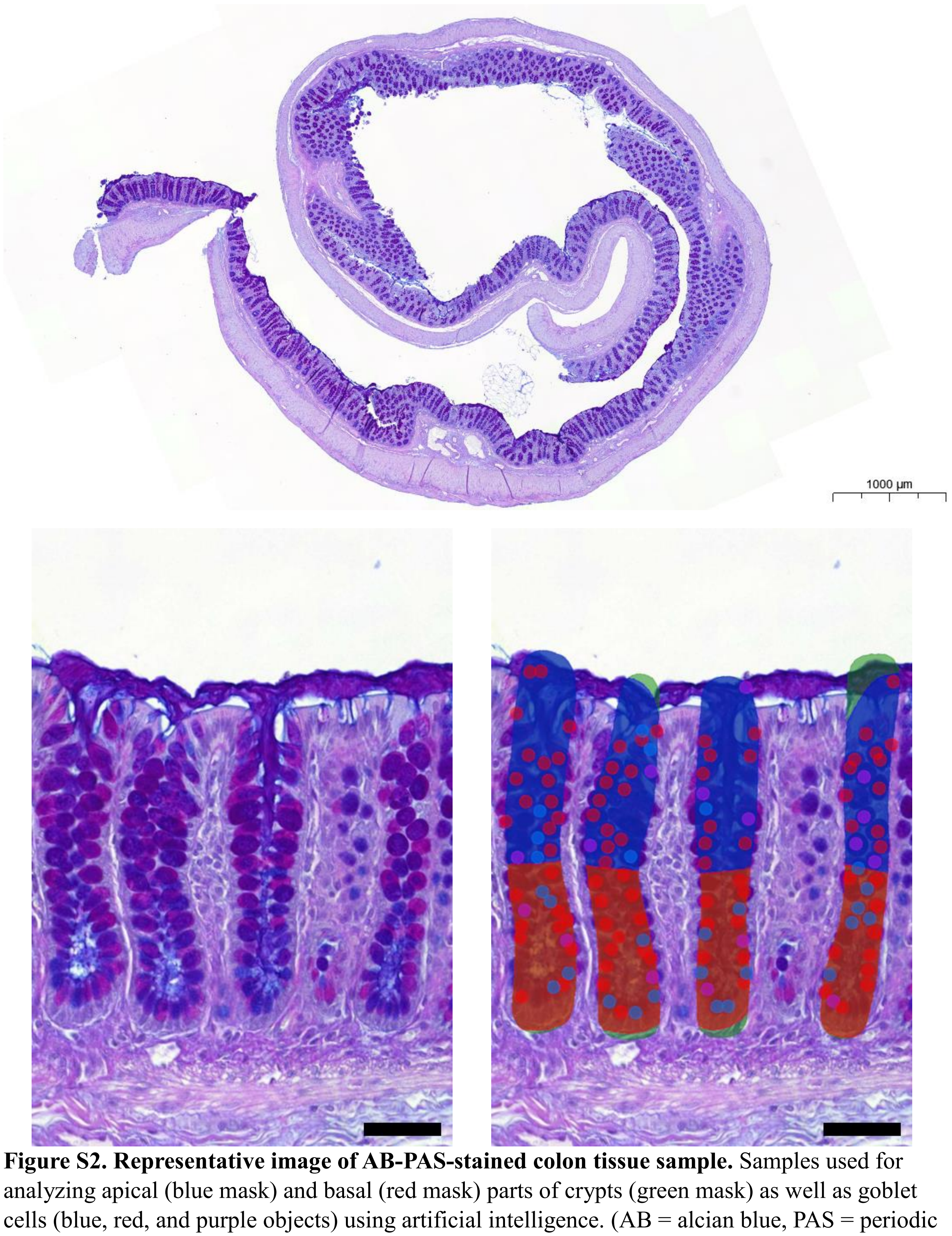

Figure S2. Representative image of AB-PAS-stained colon tissue sample. Samples used for analyzing apical (blue mask) and basal (red mask) parts of crypts (green mask) as well as goblet cells (blue, red, and purple objects) using artificial intelligence. (AB = alcian blue, PAS = periodic acid Schiff, scale bar in the second picture is 50μm)
